# Sulfur-doped activated carbon for the efficient degradation of tetracycline with persulfate: Insight into the effect of pore structure on catalytic performance†

**DOI:** 10.1039/d3ra08958d

**Published:** 2024-04-10

**Authors:** Yaoping Guo, Yaxiong Huang, Yifan Li, Yan Luo, Keng Xuan, Yadan Guo, Hao Jiang, Rui Fang

**Affiliations:** a School of Water Resources Environmental Engineering, East China University of Technology Nanchang 330013 China xuankeng@ecut.edu.cn +86 18734907983; b School of Surveying and Mapping and Spatial Information Engineering, East China University of Technology Nanchang 330013 China; c Jiangxi Engineering Province Engineering Research Center of New Energy Technology and Equipment, East China University of Technology Nanchang 330013 China

## Abstract

Sulfur-doped activated carbon has proved to be a promising metal-free catalyst for persulfate (PDS) catalytic activation for the oxidation of aqueous refractory organics. Herein, sulfur-doped porous carbon (ACS) catalysts with different pore structures and doped-S contents were prepared *via* a template method using d(+)-glucose as the carbon source, sulfur as the sulfur source, and nano-MgO with different particle sizes as templates. Characterization results showed that the particle size of MgO significantly affects the pore structure and doped-S content of ACSs catalysts: a sample synthesized with 20 nm MgO as template (ACS-20) presented the highest content of doped-S and a mesoporous structure, which endowed it with superior adsorption and catalytic performance toward tetracycline (TC) removal. The effect of catalyst dosage, TC concentration, PDS concentration and solution pH on TC removal efficiency were evaluated. The reaction mechanism, investigated by combination of EPR, quenching experiments and LC-MS, indicated that the reactive species included HO·, SO_4_˙^−^, and ^1^O_2_, but that ^1^O_2_ played the dominant role in TC oxidation through a non-radical oxidation pathway. In addition, the reusability and regeneration properties of the ACS-20 catalyst were also studied. This work provides a promising strategy and some theoretical basis for the design and preparation of activated carbon catalysts for advanced oxidation reactions from the viewpoint of pore structure design and S-doping.

## Abstract 2

Sulfur-doped activated carbon has proved to be a promising metal-free catalyst for persulfate (PDS) catalytic activation for the oxidation of aqueous refractory organics.

## Graphical abstract

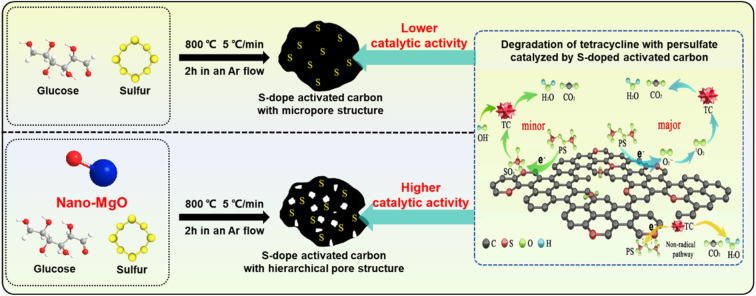

## 1. Introduction

Tetracycline (TC), the antibiotic with the second highest yield, has been extensively used for human therapy, animal/crop rehabilitation and disease prevention.^1,2^ In the past decades, large amounts of TC entered into environment through feces or urine.^3^ Generally, TC has a tetra-linked benzene ring structure, which is chemically stable and hardly to be naturally degraded by self-purification of the ecosystem, and it has been widely detected in surface water, groundwater and even drinking water.^4,5^ The exposed TC contaminant would give rise to increasing bacterial drug resistance, the generation of antibiotic genes, and eventually pose a potential risk to aquatic organisms and humans.^6^ Hence, it is crucial to exploit remediation technologies to eliminate residual TC in water.

To date, various technologies, including physical adsorption, biological degradation and advanced oxidation processes (AOPs), have been developed for the treatment of TC in water.^7^ Among these technologies, an AOP based on persulfate (PDS)/peroxymonosulfate (PMS) has attracted considerable attention in refractory organic wastewater treatment due to its advantages of high efficiency, wide pH-adaptive range and less flocculation production.^8,9^ In the last few years, the development of strategies for the generation of reactive oxygen species from PDS/PMS has been widely investigated. In particular, the investigation of PDS/PMS activation using carbonaceous materials, such as activated carbon (AC), carbon nanotubes (CNTs), graphene oxide (GO), reduced graphene oxide (rGO), and nanodiamonds (NDs), has been propelled to the forefront,^10–12^ mainly benefiting from their excellent properties, including cost-effectiveness, environmental benignity, abundant surface functional groups, and highly developed pore structure.

A large number of studies have shown that carbonaceous materials doped with heteroatoms such as N, S, B, or P, can be applied as promising alternatives to metal-based catalysts due to their remarkable catalytic efficiency.^13–15^ In our previous studies,^16,17^ several sulfur-doped activated carbons (S-ACs) have been successfully synthesized by chemical activation method with KOH and K_2_CO_3_ as activators, and the S-ACs exhibited excellent catalytic activity toward PDS activation for phenol and *p*-chlorophenol degradation. The excellent catalytic activity of the S-ACs was mainly attributed to the fact that sulfur doping improved the hydrophilicity of activated carbon, changed the spin density of neighboring carbon atoms, and enhanced the catalytic activity of activated carbon.^18^ However, the pore structures of the above-mentioned S-ACs mainly consisted of micropore structures, which showed inferior adsorption performance and catalytic activity toward TC owing to its large molecular structure. Therefore, it is speculated that sulfur-doped activated carbon with a mesoporous structure might present superior performance for TC removal.

The well-known template method has commonly been used for the preparation of activated carbon with a mesoporous structure, and this pore structure could be easily tuned by changing the species and the particle size of the templating agent.^19^ By using magnesium oxide as a template for the preparation of mesoporous carbon materials, it is possible to obtain porous carbon with a precisely controllable pore structure directly from thermoplastic carbon precursors. The significant advantage of this method is that it does not rely on any additional stabilization or activation steps.^20^ In addition, the adjustment of the pore structure becomes simple and convenient by changing the type of template and adjusting its particle size. Therefore, it is reasonable to suppose that the particle size of the template would have a significant effect on the precursor carbonization process, including the development of porous structures and the doping level of sulfur, that in turn would affect its catalytic oxidation performance. However, research into how the particle size of the template affects the pore structure as well the catalytic properties of sulfur-doped activated carbons toward the degradation of TC with persulfate, is still unclear.

Hence, in this study, sulfur-doped activated carbon (ACS) catalysts were prepared *via* a template method using nano-MgO as the template. The effects of nano-MgO particle size on pore structure as well as sulfur doping level of the ACSs and their catalytic performance towards TC removal were investigated. The operating parameters of the effects, including TC concentration, catalyst loading, PDS concentration, and solution pH, were evaluated. In addition, by combining the results of quenching experiments, EPR and LC-MS, the possible PDS activation and TC degradation mechanisms over ACS were conjectured. Finally, the reusability of the ACSs was also studied. The results of this work could provide an effective technology for the future development of efficient sulfur-doped activated carbons for TC wastewater treatment.

## 2. Materials and methods

### 2.1 Chemicals and reagents

The precursors d(+)-glucose (C_6_H_12_O_6_, 99%) and sulfur (S, 99.9%) for catalyst preparation were purchased from Macklin. NaOH (AR, 96%), tetracycline (TC, CP), l-histidine (C_6_H_9_N_3_O_2_, 99%) and potassium persulfate (K_2_S_2_O_8_, 99.5%) were obtained from Aladdin. Hydrochloric acid (HCl, AR) and methanol (MeOH, 99.7%) were obtained from Xilong Scientific Co., Ltd 20 nm nanoMgO was obtained from Xianfeng Nanomaterials Technology Co., Ltd 50 nm nanoMgO was obtained from Macklin. All chemicals were used as received. All solutions were prepared with ultrapure water.

### 2.2 Preparation of ACSs

**Scheme 1:**
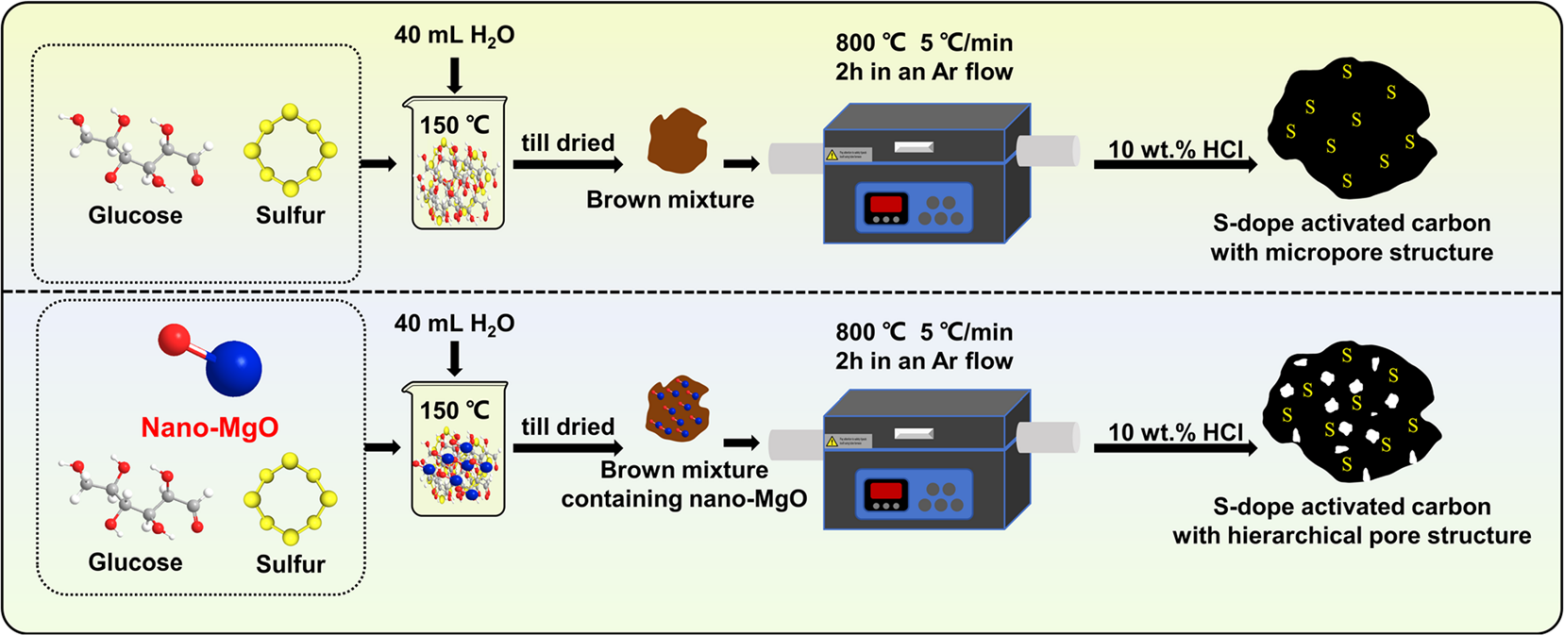
Schematic diagram of the preparation procedure of ACSs catalysts.

S-doped activated carbons (ACSs) with different pore structures were prepared *via* a template method using nano-MgO with different particle sizes (∼20 nm and ∼50 nm) as the template (Scheme 1). Briefly, 2 g of glucose, 0.05 g of S powder and 1.0 g of nano-MgO were mixed evenly in 40 mL of water, then the mixture was stirred in an oil bath at 150 °C until it dried. Thereafter, the obtained product (named the C–S precursor) was carbonized in a tubular furnace at a heating rate of 5 °C min^−1^ from room temperature to 800 °C and held for 2 h in an Ar flow. After naturally cooling to room temperature, the obtained product was washed with 10 wt% HCl solution and ultrapure water several times to remove the nano-MgO template, followed by drying in an oven at 110 °C overnight for further use. The as-made samples were named ACS-20 and ACS-50 according to the particle size of nano-MgO. For comparison, a sample without the addition of nano-MgO was prepared under the same conditions (denoted ACS-0).

### 2.3 Characterizations of ACSs

The morphologies of the samples were characterized using a scanning electron microscope (SEM, QUANTA FEG 450). All the samples were coated with a thin gold film under a vacuum before microscopy. The elemental contents of the carbon samples were determined using a Vario EL Cube apparatus with the oxygen content calculated by mass difference. The chemical composition of the materials was analyzed *via* an X-ray photoelectron spectrometer (XPS, Escalab 250Xi) using an Al Ka monochromatic source (1486.6 eV). The C 1s peak at 284.8 eV was taken to calibrate the binding energies of the XPS spectra. The specific surface area and pore structure parameters of the samples were determined using a specific surface area and porosity analyzer (3Flex 5.02). The functional groups contained on the surface of the material were characterized using FT-IR on a Thermo Scientific Nicolet iS20, USA, and the scanning wavelength range was 500–4000 cm^−1^. To investigate the mechanism of the TC degradation process, an electron paramagnetic resonance (EPR) test and free radical quenching experiments were performed. EPR was performed on a Bruker EMS-plus instrument, with 5,5-dimethyl-1-pyrroline N-oxide (DMPO) as a spin-trapping agent for SO_4_˙^−^ and HO˙, and 2,2,6,6-tetramethylpiperidine (TEMP) for ^1^O_2_.

### 2.4 Catalytic performance evaluation

The batch catalytic oxidation of TC was carried out in a 250 mL conical flask containing 200 mL of TC solution (50 mg L^−1^). The flask was shaken at 220 rpm in a constant-temperature shaker (SHA-C) at 25 °C. Typically, 0.01 g of ACS was dispersed in the TC solution and shaken for 30 min, then 2 mmol L^−1^ of PDS was added to initiate the catalytic oxidation process (PDS/TC mole ratio = 17.8). At predetermined time intervals, 5 mL of reaction solution was taken out with a syringe, and analyzed using a UV-visible spectrophotometer at 357 nm after passing through a 0.22 μm filter membrane. After each run, the passivated carbons were recycled and washed with water and methanol several times, followed by drying in an oven at 110 °C overnight for reuse tests. In addition, the effects of catalyst loading, TC concentration, PDS concentration, and solution pH on TC oxidation were also investigated. The pH of the solution was adjusted by adding 0.1 M HCl or NaOH. All the experiments were performed in duplicate and the average values of the data are presented.

### 2.5 Detection and calculation of TC concentration

The concentration of TC was measured using a UV-visible spectrophotometer with wavenumber at 357 nm, and the removal rate of TC was calculated with eqn (1), where *η* is the degradation rate of TC, *c*_0_ is the concentration of TC at the initial reaction time and *c* is the concentration of TC at the given reaction time.1
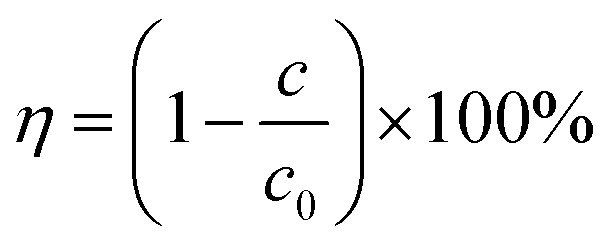


To investigate the adsorption process for TC, a pseudo-first-order adsorption kinetic model, a pseudo-second-order adsorption kinetic model and an intra-particle diffusion kinetic model were used to fit the experimental data. The kinetic model formulae are shown in eqn (2)–(4), where *q*_*t*_ is the adsorption amount at time *t*, and *k*_f_, *k*_s_, and *k*_i_ are the adsorption rate constants of the pseudo-first-order, pseudo-second-order and intra-particle diffusion kinetic models, respectively.

2 *q*_*t*_ = *q*_e_(1 − e^−*k*_f_*t*^)

3
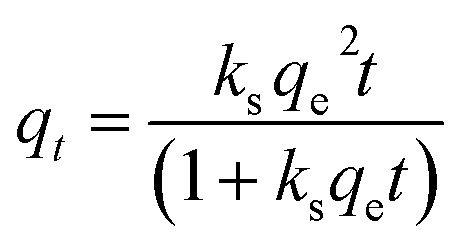

4 *q*_*t*_ = *k*_i_*t*^0.5^ + *C*

## 3. Results and discussion

### 3.1 Characterization of the ACSs

The contents of C, H, O, S and N in the C–S precursors were calculated according to the amount of glucose and S powder, and the elemental compositions of the ACS catalysts were determined using an elemental analyzer (EA). As shown in Table 1, the contents of C, H, O, S, N in the C–S precursor were 39.03%, 6.50%, 52.03%, 2.44% and 0%, respectively. While for all the ACS catalysts, the contents of H, O and S dramatically decreased, the content of C exceeded 80%. This was probably because volatile components, including H_2_O, H_2_S and organic decomposition products, were generated during the carbonization process. In addition, nano-MgO could also react with S element to form magnesium sulfide, magnesium sulfite, or magnesium sulfate, which would be removed in the catalyst washing process.^21^ The S content of ACS-0 synthesized without nano-MgO as template was only 0.77%, which increased to 3.54% for ACS-20, indicating that the nano-MgO template was beneficial for sulfur doping. However, the S content of ACS-50 nano-MgO decreased to 1.43% as the particle size of the nano-MgO increased to 50 nm. This is probably because in the heat treatment process, the C–S precursor would firstly undergo thermal cracking to lose hydrogen and oxygen elements to form a carbon skeleton; then the carbon skeleton and sulfur elements would react to form C–S bonds, *i.e.* the element S was doped into the carbon skeleton.^22^ As is well known, the addition of a nano-MgO template can induce the formation of a pore structure in activated carbon, which could offer more reactive space for the reaction of the carbon skeleton and S, thus promoting the doping of S element. Based on the above analysis, the lower S-doping level of ACS-50 is probably due to the smaller reactive surface area that the nano-MgO with particle size of 50 nm can provide at the same mass.^23^

**Table 1:** The elemental composition of C–S precursor and ACS samples

Sample	Element content (wt%)				
	C	H	O^a^	S	N
C–S precursor	39.03	6.50	52.03	2.44	0^b^
ACS-0	90.91	0.80	7.36	0.77	0.16
ACS-20	82.92	0.88	12.53	3.54	0.13
ACS-50	84.83	0.97	12.64	1.43	0.13

a The content of O was calculated by mass difference.^24^

b N in the C–S precursors was calculated according to the amount of glucose and S powder; thus the value is zero.

**Fig. 1:**
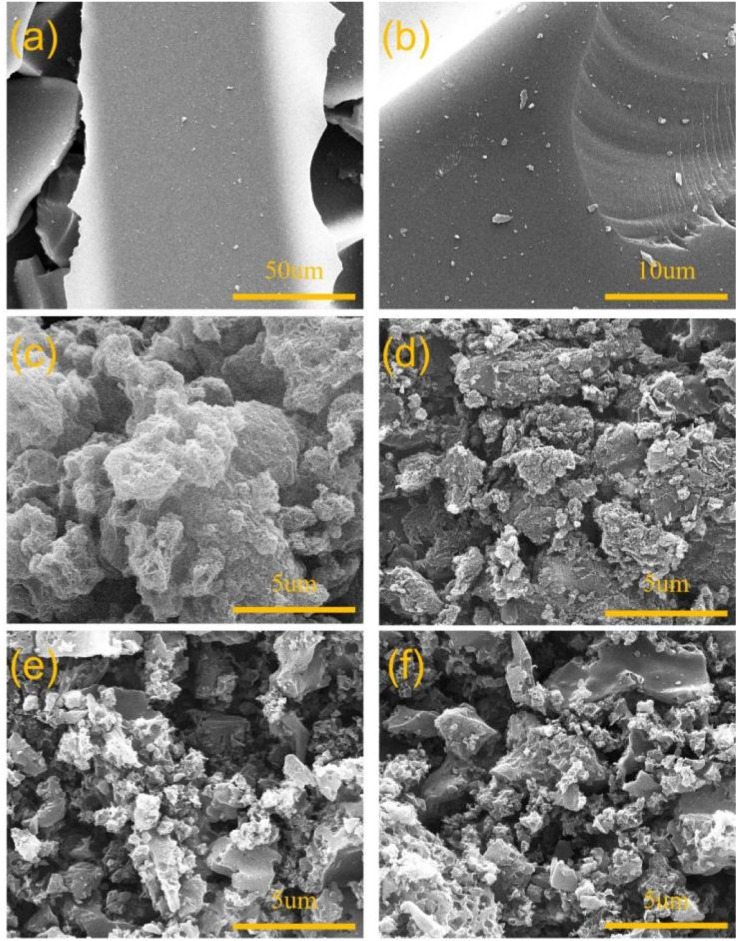
The SEM images of (a and b) ACS-0, (c and d) ACS-20, and (e and f) ACS-50.

The effects of nano-MgO on the specific surface area and pore structure of the ACSs were analyzed by a combination of SEM and N_2_ isothermal adsorption–desorption. As can be seen from the SEM images presented in Fig. 1a and b, the surface of ACS-0 without a nano-MgO template was smooth and almost no pores could be observed. While, it was found from Fig. 1c–f that the morphologies of ACS-20 and ACS-50 were rough and fragmented. In addition, as shown in Fig. S1a,† in the low-pressure region, the N_2_ adsorption increased rapidly and tended to saturate. As the pressure further increased, the N_2_ adsorption barely increased and then showed a clear plateau, *i.e.*, the adsorption–desorption curve belongs to typical type-I isotherm, suggesting the mainly microporous structure of ACS-0. For ACS-20, a hysteresis loop was observed due to the presence of a mesoporous structure, which was also confirmed by the pore size distribution presented in Fig. S1b.† Compared with ACS-20, the pore structure of the ACS-50 sample predominantly consisted of micro-mesopores with a type-IV isotherm,^25^ the pore sizes of which were mainly distributed in the range 1.5–2.5 nm (Fig. S1b†). These results clearly indicated that the nano-MgO template facilitated the formation of mesoporous structures in the ACS-20 and ACS-50 catalysts.

According to Table 2, only a specific surface area (*S*_BET_) of 388 m^2^ g^−1^ and total pore volume (*v*_p_) of 0.14 cm^3^ g^−1^ with an average pore size of 1.48 nm were detected in ACS-0, but the addition of a nano-MgO template promoted the formation of porosity and surface area. Remarkably, ACS-20 exhibited the highest *S*_BET_ of 889 m^2^ g^−1^ and total pore volume (*v*_p_) of 1.99 m^3^ g^−1^, but the values for ACS-50 were decreased. As mentioned before, nano-MgO can act as template for the formation of pore structure in carbon materials. The formed surface area can subsequently provide space for the reaction between S and oxygen-containing functional groups of the C–S precursor, which could additionally induce the formation of more pore structure. Therefore, for the same mass, nano-MgO with a smaller particle size can provide more particles as a template for the formation of a pore structure. The formed pore structure can also provide more space for the reaction between S and oxygen-containing functional groups of the C–S precursor, thus inducing the formation of new larger pores and average pore sizes in ACS-20.

**Table 2:** Textural properties of the ACSs catalysts

Sample	BET surface area *S* (m^2^ g^−1^)	Total pore volume *v* (cm^3^ g^−1^)	Micropore volume *v* (cm^3^ g^−1^)	Average pore size *d* (nm)
ACS-0	388	0.14	0.13	1.48
ACS-20	889	1.99	0.02	8.96
ACS-50	537	0.86	0.10	2.99

The XPS spectroscopic technique was performed to investigate the surface chemistry of the ACS samples and the results are presented in Fig. S2.† The peaks at 164.0 eV and 165.2 eV correspond to the S 2p_1/2_ and S 2p_3/2_ spectra of –C–S–C–, respectively. While the peaks at 168.4 eV and 169.5 eV correspond to the S 2p_1/2_ and S 2p_3/2_ spectra of –C–SO_*x*_–C– (*x* = 2, 3, 4), respectively.^26,27^ Previous studies confirmed that –C–SO_*x*_–C– was an acidic functional group in sulfur-doped activated carbon, which could increase the surface acidity of activated carbon and was not favorable for electron transfer, while the S atom in –C–S–C– is the dominant active site for PDS activation attributed to the fact that the S atom could change the spin density of neighboring carbon atoms to improve the electron-donating ability of activated carbon.^16,28^ From Fig. S2,† it was found that the existence of S on the surface of the ACSs is mainly in the form of –C–S–C–, and the percentage of –C–S–C– follows the order ACS-20 > ACS-50 > ACS-0, indicating that using nano-MgO with a particle size of 20 nm is more favorable for the formation –C–S–C–.

### 3.2 Catalytic performance of the ACSs

#### 3.2.1 Effect of nano-MgO particle size on TC removal efficiency of the ACSs

**Fig. 2:**
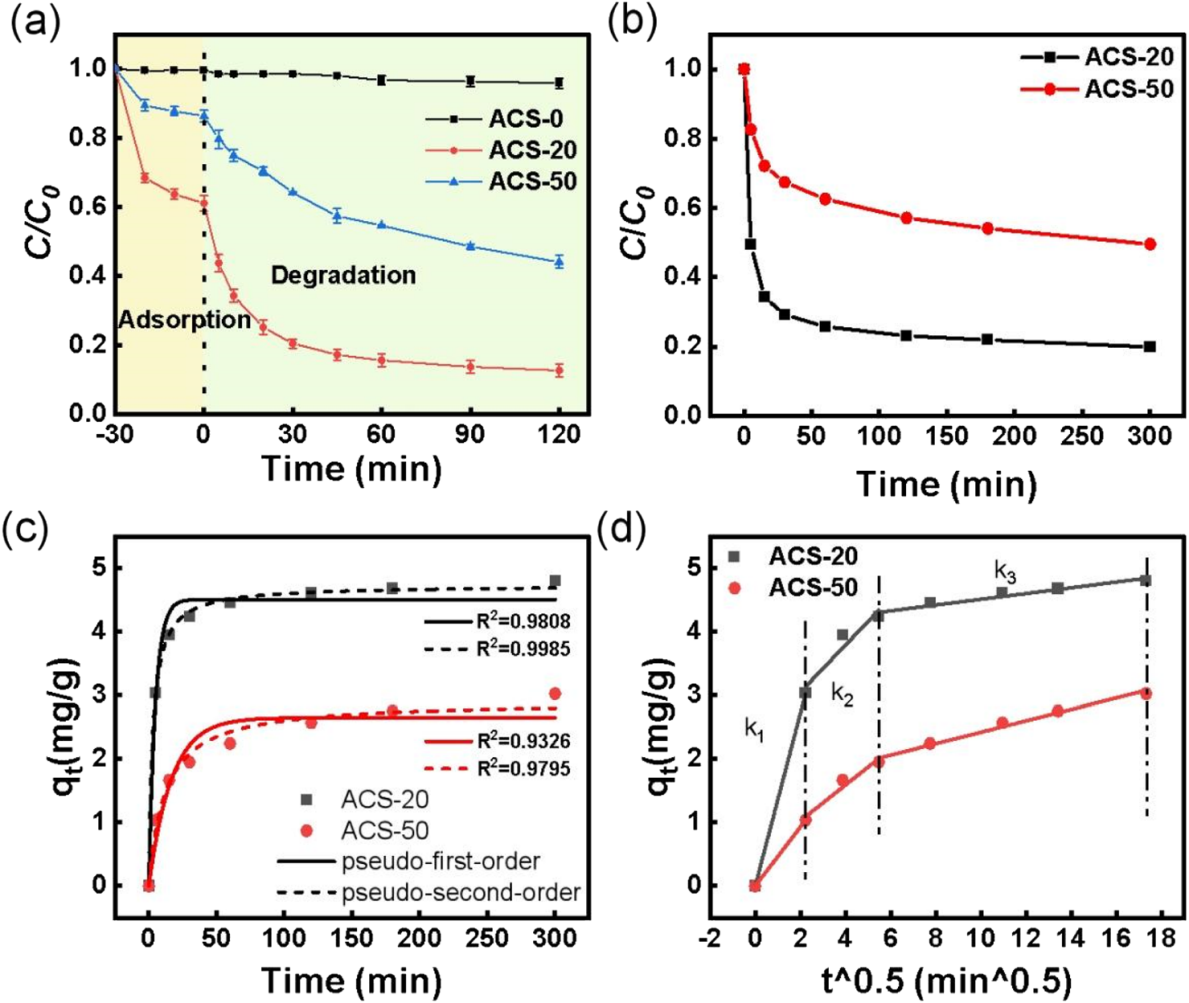
(a) Removal of TC over different reaction systems; reaction conditions: [TC] = 50 mg L^−1^, [catalyst] = 0.05 g L^−1^, [PDS] = 2 mM, and temperature = 25 °C. (b) Adsorption kinetics of TC by ACS catalysts; reaction conditions: [TC] = 30 mg L^−1^, [catalyst] = 0.05 g L^−1^ and temperature = 25 °C. (c) Adsorption kinetics fitted with pseudo-first-order and pseudo-second-order models; (d) intra-particle diffusion kinetic models of ACS-20 and ACS-50.

The effect of nano-MgO particle size on the TC removal efficiency of the ACSs was investigated *via* adsorption–oxidation batch tests. As shown in Fig. 2a, the adsorption efficiencies for TC on ACS-0, ACS-20 and ACS-50 were 0.5%, 38.9% and 13.5%, respectively. According to the N_2_ adsorption–desorption results, although the specific surface area of ACS-0 reached 388 m^2^ g^−1^, its pore structure mainly exists in the form of micropores and the average pore size was only 1.48 nm, leading to difficult adsorption of TC molecules with their large diameter. Compared with ACS-0, ACS-50 with its higher specific surface area and larger average pore size (2.99 nm) showed better adsorption performance. ACS-20 with the largest specific surface area and the largest average pore size (8.96 nm) presented the best adsorption performance. Generally, the specific surface area of activated carbon played a vital role in its adsorption performance, and the adsorption capacity usually increased with *S*_BET_. To assess the effect of *S*_BET_ on the adsorption capacity in this work, the adsorption capacity per unit of specific surface area (*q*_e_/*S*_BET_) of the ACSs was calculated. The *q*_e_/*S*_BET_ values of ACS-0, ACS-20 and ACS-50 were 0.013, 0.438, and 0.251 mg m^−2^, respectively, indicating that the specific surface area might not be the dominant factor in determining its adsorption performance. Hence, we hypothesize that the adsorption performance of the ACSs might also be influenced by the pore structure, since the internal diffusion resistance of the TC molecule with its large diameter in mesopores was much lower than that of micropores, which will be further discussed in the section on adsorption kinetics. The above analysis implied that the particle size of nano-MgO has an important effect on the pore structure of the ACSs, which in turn influences its adsorption performance.

After PDS was added into the reaction systems, ACS-20 showed the best performance with 87.2% TC removal in 120 min, and ACS-50 displayed moderate PDS activity with 55.9% TC removal. While only 4.2% TC removal was achieved on ACS-0. According to the previous adsorption performance analysis, the TC adsorption ability as well as the PS-activation ability of ACS-0 were extremely low, mainly due to its microporous structure as well as the low concentration of –C–S–C groups. Notably, both the adsorption performance and the catalytic activity of ACS-20 were higher than those of ACS-50. In previous studies,^29,30^ it has been proved that higher adsorption capacity would lead to a more rapid degradation process, mainly attributed to the fast contact between the adsorbed contaminants and the reactive species. In order to evaluate the oxidation ability, the respective adsorption contributions were deduced. The results showed that about 48.3% and 42.4% of extra TC was removed on ACS-20 and ACS-50, respectively, suggesting that the ACS-20 sample exhibits the highest catalytic activity not only due to its excellent pore structure, but also benefiting from its excellent chemical characteristics. Our previous studies confirmed that the thiophene sulfur (–C–S–C–) functional groups in sulfur-doped activated carbon were important active sites for PDS activation.^16,28,31^ Combined with the XPS results, it can be seen that ACS-20 presents a higher content of –C–S–C– functional group than ACS-50, thus endowing it with higher catalytic performance.

To further understand the effect of pore structure on the catalytic performance of the ACSs, the adsorption kinetics of TC by ACS-20 and ACS-50 were also analyzed. The adsorption kinetics were fitted by pseudo-first-order adsorption and pseudo-second-order adsorption models, respectively. From Fig. 2b and c, it was found that the adsorption processes of TC by ACS-20 and ACS-50 were more in line with the pseudo-second-order kinetic model, indicating that the adsorption of TC by ACS-20 and ACS-50 is chemical adsorption. In addition, a Weber–Morris intra-particle diffusion model was employed to analyze the diffusion process of TC. As shown in Fig. 2d and Table S1,†*k*_1_, *k*_2_ and *k*_3_ represent the diffusion rate constant at the stages of film diffusion, internal diffusion, and dynamic balance, respectively. The values of *k*_1_ and *k*_2_ of ACS-20 are both greater than those of ACS-50. Wherein, the bigger *k*_1_ of ACS-20 might benefit from its rich surface adsorption groups, and the larger *k*_2_ of ACS-20 might be attributed to its large pore size, as reflected by the N_2_ adsorption–desorption results.

Based on the above analysis, it is clear that the pore structure is the main factor affecting the adsorption properties of the ACSs, while the pore structure as well as the sulfur-containing functional groups together affected the PDS activation and TC oxidation catalytic properties of the ACSs. ACS-20, with a larger specific surface area, larger pore size and the highest thiophene-sulfur functional group content, exhibited the best adsorption and catalytic properties, and was chosen for further tests in subsequent studies.

#### 3.2.2 Effects of reaction parameters on TC removal

**Fig. 3:**
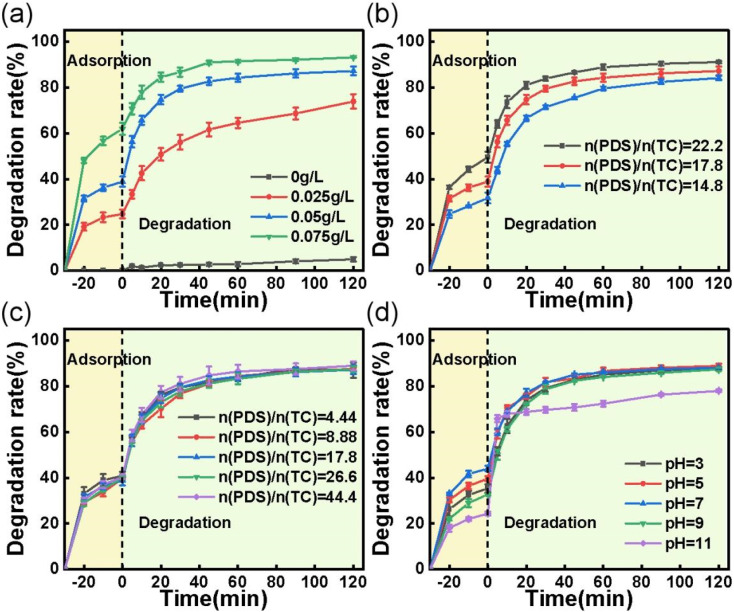
Effect of (a) catalyst dosage; reaction conditions: [TC] = 50 mg L^−1^, [PDS] = 2 mM, and temperature = 25 °C. (b) Effect of TC concentration; reaction conditions: [catalyst] = 0.05 g L^−1^, [PDS] = 2 mM, and temperature = 25 °C. (c) Effect of PDS concentration; reaction conditions: [TC] = 50 mg L^−1^, [catalyst] = 0.05 g L^−1^, and temperature = 25 °C. (d) Effect of initial pH on the adsorption and degradation of TC; reaction conditions: [TC] = 50 mg L^−1^, [catalyst] = 0.05 g L^−1^, [PDS] = 2 mM, and temperature = 25 °C.

The impacts of several reaction parameters, including catalyst loading, TC concentration, PDS concentration, and initial solution pH, were investigated using the best performing ACS-20/PDS system. As illustrated in Fig. 3a and b, the TC removal efficiency significantly increased with an increase in ACS-20 dosage and decreased with an increase in TC concentration, mainly because the higher catalyst dosage could provide a larger specific surface for the TC adsorption area and more active sites for the generation of active species for TC degradation. Whereas, for the degradation of TC with higher concentration, the active sites and the amount of PDS were relatively insufficient, which led to a decrease in TC removal efficiency.

PDS concentration is also an important parameter that affects the degradation of organics. As shown in Fig. 3c, only a slight increase in TC degradation rate was observed when the PDS concentration increased from 0.5 to 5 mM. It is conjectured that PDS activation and TC degradation are surface processes, whereas the formation of reactive species from PDS and the adsorption–desorption of TC and its intermediates are diffusion- and equilibrium-driven processes. At an ACS-20 dosage of 0.05 g L^−1^, a larger surface area and more active sites are available for TC adsorption and PDS activation, resulting in the excess generation of intermediates. In this case, desorption of the intermediates was critical for controlling the degradation rate, leading to a minimal effect from PDS concentration.^31^

The effect of initial solution pH (pH 3–11) on TC removal was also investigated. As can be seen from Fig. 3d, the adsorption efficiency for TC increased as the pH increased from 3 to 7, and then decreased as the pH increased further. The variation in adsorption efficiency for TC was probably related to the states in which TC molecules were present in solution. Maged *et al.* reported that TC mainly existed in cationic (TCH^3+^) form under strongly acidic conditions (pH < 3.3), in amphoteric ion (TCH^2±^) form under weakly acidic conditions (3.3 < pH < 7.7), and then in anionic form when pH > 7.7.^32^ According to the reports of Pang *et al.* and Qi *et al.*,^33,34^ the surface potential of S-doped activated carbon would become positively charged and negatively charged under strongly acidic and basic conditions, respectively. The surface zero charge point of ACS-20 was determined to be pH_pzc_ = 5.3 using the method reported by Sergio Morales-Torres (the specific method is described in Text S1†).^35^ According to the FT-IR characterization (Fig. S3†), the surface of ACS-20 contains the groups –COO^−^, –C–O–C– and –OH, which are acidic and could deprotonize in the solution at pH > 5.3, and the surface of ACS-20 would become negatively charged, thereby exhibiting electrostatic repulsion toward the negatively charged TC species, and leading to the lower adsorption efficiency for TC. In addition, it was noted that TC degradation was almost uninfluenced by pH when the solution was below 9; *i.e.* ACS-20 showed excellent catalytic activity toward PDS over a wide pH range. However, it was noted that the TC degradation efficiency significantly decreased as the pH increased to 11, which was probably because the reactions between reactive oxidation species including SO_4_˙^−^ and HO˙ with OH^−^eqn (5)–(8) under strongly basic conditions were enhanced due to the high concentration of OH^−^.^36,37^

5 SO_4_˙^−^ + H_2_O → SO_4_^2−^ + HO˙ + H^+^, *k* < 6 × 10^1^ M^−1^ s^−1^

6 SO_4_˙^−^ + OH^−^ → SO_4_^2−^ + HO˙, *k* = 7.3 × 10^7^ M^−1^ s^−1^

7 HO˙ + OH^−^ → O˙^−^ + H_2_O, *k* = 1.2 × 10^10^ M^−1^ s^−1^

8 SO_4_˙^−^ + HO˙ → HSO_4_^−^ + 0.5O_2_, *k* = 1−10 × 10^9^ M^−1^ s^−1^

### 3.3 PDS activation mechanism over ACS-20

**Fig. 4:**
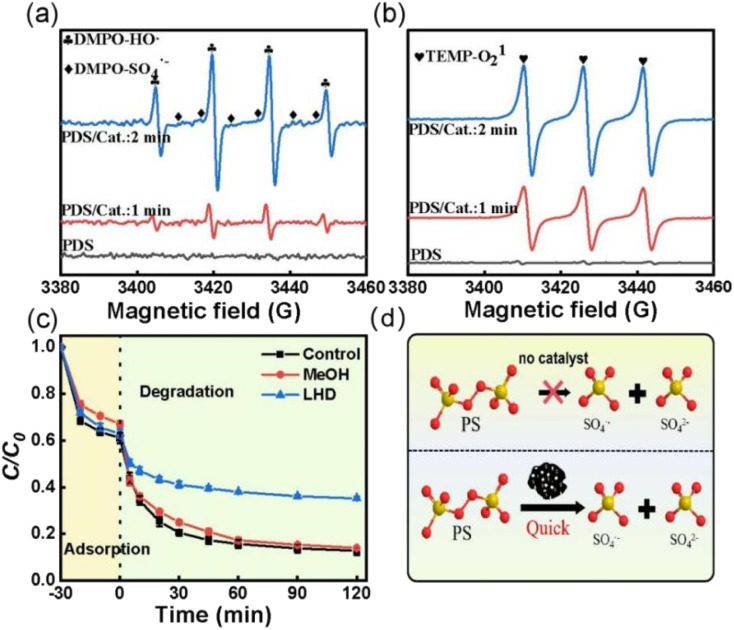
The EPR spectra with (a) DMPO and (b) TEMP as the trapping agent. (c) Effect of different capture agents on the removal of TC; reaction conditions: MeOH: 2000 mM, LHD: 30 mM, [TC] = 50 mg L^−1^, [catalyst] = 0.05 g L^−1^, [PDS] = 2 mM, and temperature = 25 °C. (d) Schematic diagram of PDS activation.

To investigate the PDS activation mechanism over ACS-20, electron paramagnetic resonance (EPR) tests and free radical quenching experiments were performed. From Fig. 4a, it was found that no characteristic peaks of DMPO-HO· or DMPO-SO_4_˙^−^ appeared when PDS was added alone, while both DMPO-HO˙ and DMPO-SO_4_˙^−^ characteristic peaks were detected as both ACS-20 and PDS were added into the system, and the peak intensity of the characteristic peaks gradually increased as the reaction time was prolonged from 1 min to 2 min, indicating that ACS-20 could effectively and continuously activate PDS to generate HO· and SO_4_˙^−^ (Fig. 4d). Fig. 4b shows that ^1^O_2_ was generated in the ACS/PDS system, and the amount of ^1^O_2_ gradually increased with reaction time. The EPR results clearly demonstrated the simultaneous presence of HO˙, SO_4_˙^−^, and ^1^O_2_ reactive species in this reaction system.

To further identify which active species played the dominant roles in TC degradation, trapping experiments were conducted with methanol (MeOH) and l-histidine (LHD) as free radical scavengers,^38^ where MeOH was mainly used to trap HO· and SO_4_˙^−^ produced in the ACS/PDS system, and LHD was mainly used to trap ^1^O_2_ in the solution. Fig. 4c shows that the degradation rate of TC was slightly decreased when MeOH was added to the system. In contrast, the degradation efficiency for TC significantly decreased when LHD was added to the system, indicating that, although active species such as HO˙, SO_4_˙^−^ and ^1^O_2_ were generated in the system, ^1^O_2_ played the dominant role. In addition, it was noted that, although the addition of 30 mM LHD inhibited the TC removal efficiency, about 60% of TC was removed, suggesting that non-radical pathways other than ^1^O_2_ might also participate in this reaction.

### 3.4 Tetracycline degradation mechanism over ACS-20

**Fig. 5:**
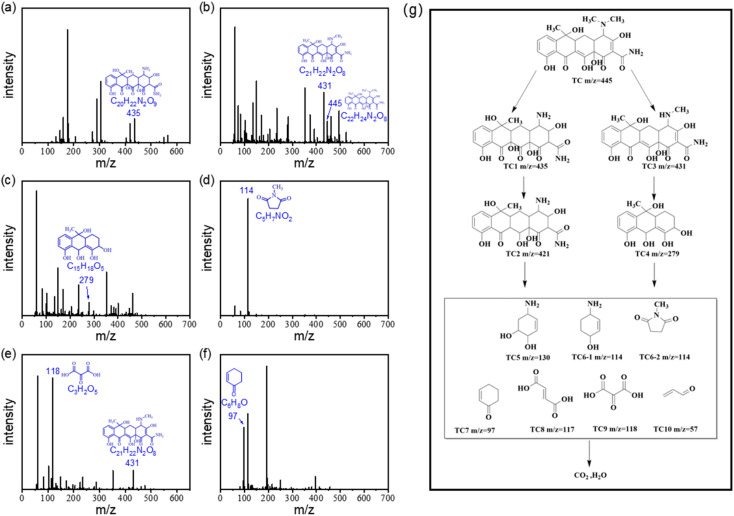
(a) to (f) LC/MS mass spectrum and the molecular structure analysis of intermediates for TC degradation in the ACS-2/PDS system. (g) Plausible degradation pathways for TC.

To investigate the TC degradation mechanism in the ACS-20/PDS system, the intermediates generated in the reaction system were also detected using a liquid mass spectrometer (LC-MS, Ultimate 3000 UHPLC-Q Exactive). By screening the mass spectrometry data, the possible degradation intermediates of TC were identified and are presented in Fig. 5a to f. Based on the LC-MS results, possible degradation pathways of TC were proposed and are presented in Fig. 5g. Briefly, in the molecular structure of TC, *N*-demethylation occurs on the N–C bond, and C=C is attacked by radicals through hydrogenation and hydroxyl to generate TC1, followed by dehydration to form TC2.^39^ Other pathways involve a *N*-demethylation reaction on the TC molecule to generate TC3, followed by the generation of TC4 *via* ring-opening and hydroxylation of TC3.^40,41^ Then, in the ACS/PDS system, the above intermediates are oxidized *via* functional group loss and ring-opening reactions to form TC5, TC6, TC7, TC8, TC9 and TC10, which are finally oxidized to CO_2_ and H_2_O.

**Scheme 2:**
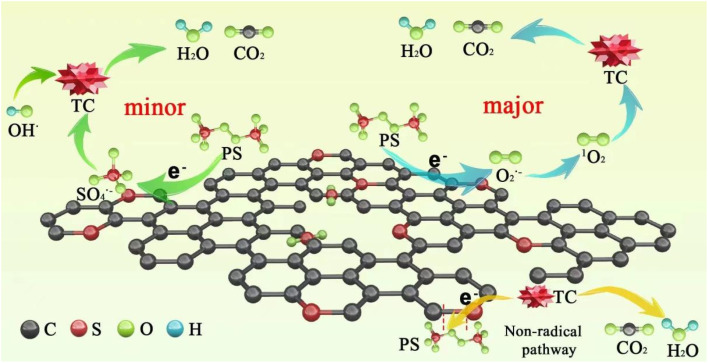
Plausible mechanism of PDS activation and TC degradation in the ACS/PDS system.

Based on the above results, a possible mechanism for TC degradation *via* ACS/PDS was proposed, as shown in Scheme 2. On the one hand, PDS obtains electrons from ACSs and is activated to form SO_4_˙^−^ through eqn (9). On the other hand, PDS is activated to form O_2_˙^−^ through eqn (10); then the generated O_2_˙^−^ is further converted into ^1^O_2_ (eqn (11) and (12)).^42^ In addition, we supposed that a metastable PDS complex might also participate in the PDS activation and TC oxidation processes, resulting from electron transfer from the carbon surface to PDS for the generation of reactive complexes.

9 S_2_O_8_^2−^ + e^−^ → SO_4_^2−^ + SO_4_˙^−^

10 2S_2_O_8_^2−^ + 2H_2_O + e^−^ → 4SO_4_^2−^ + O_2_˙^−^ + 4H^+^

11 2O_2_˙^−^ + 2H^+^ → H_2_O_2_ + ^1^O_2_

12 2O_2_˙^−^ + 2H_2_O → H_2_O_2_ + ^1^O_2_ + OH^−^

### 3.5 Reusability and stability of ACS-20

**Fig. 6:**
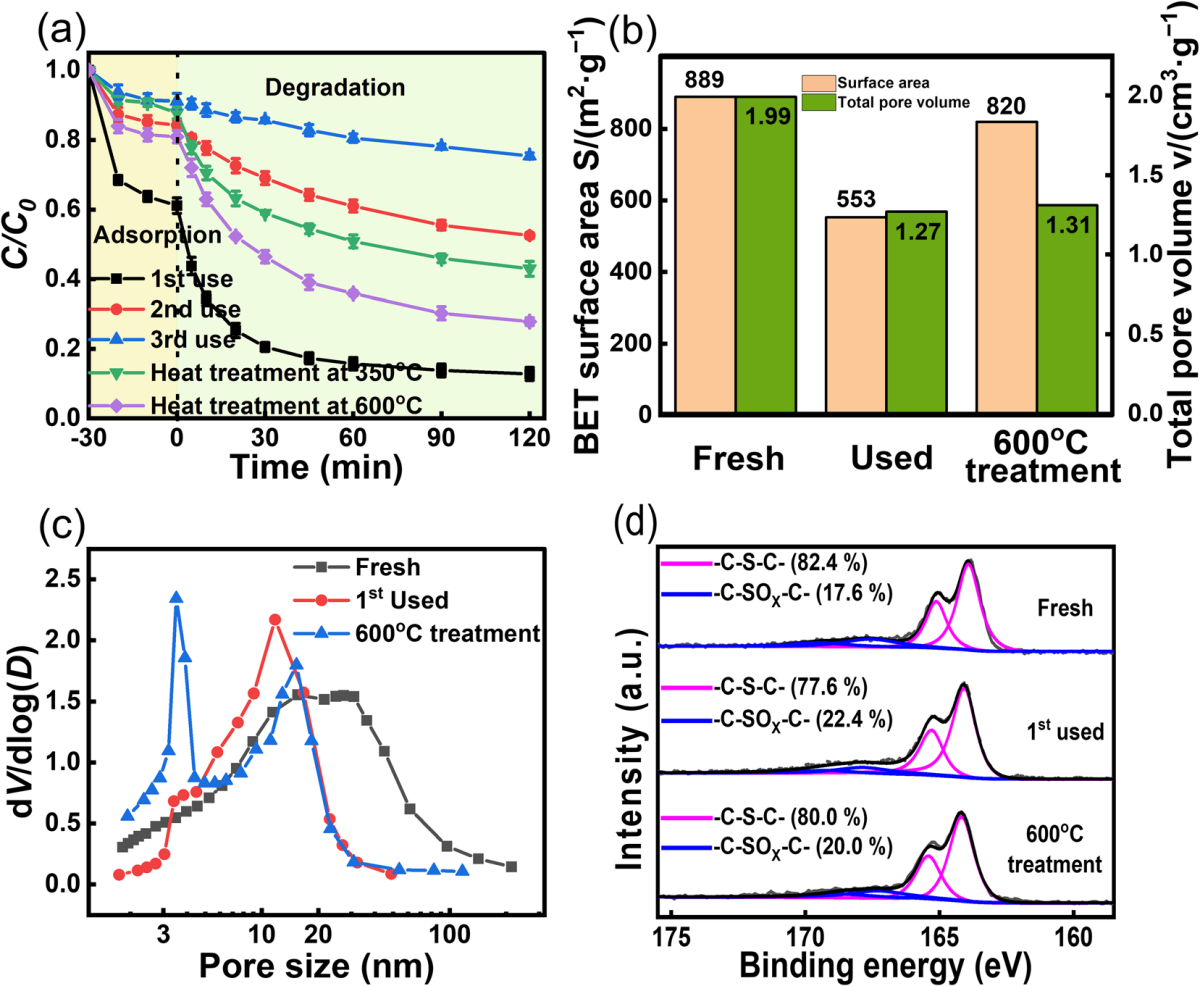
(a) Reusability and regeneration of ACS-20; (b) *S*_BET_ and total pore volume data, (c) pore size distributions and (d) the XPS S 2p spectra of fresh, used and after regeneration of ACS-20. Reaction conditions: [TC] = 50 mg L^−1^, [catalyst] = 0.05 g L^−1^, [PDS] = 2 mM, and temperature = 25 °C.

Reusability is important for assessing the potential of catalysts for practical applications.^43,44^ ACS-20 with the best activity was chosen for a reusability experiment. After each cycle, the ACS-20 catalyst was recovered and subsequently washed with deionized water and methanol several times, filtered, and dried. As shown in Fig. 6a, after the second and third cycles, TC removal decreased from 87.2% to 47.5% and 24.6%, respectively, indicating that ACS-20 was severely deactivated. A comprehensive comparison of the catalytic activity and stability of other materials for TC removal (Table 3) revealed that most activated-carbon-based materials as well as ACS-20 in this work possess poor stability in an advanced oxidation reaction system, but ACS-20 required a lower catalyst dosage and lower persulfate dosage to achieve the same desired catalytic performance.

**Table 3:** Stability comparison of reported catalysts and the ACS-20 catalyst in this work

Catalyst	Catalyst (g L^−1^)	Persulfate (mmol L^−1^ or g L^−1^)	TC (mg L^−1^)	Efficiency (%)	Stability	EF^a^ (mg g^−1^)	Ref.
OBC-Fe_3_O_4_	0.4	10 mmol L^−1^	20	92.3%	3rd >80%	46.15	45
CA/GO	0.75	10 g L^−1^	80	>98%	4th to 85.3%	104.53	46
FeS@BC	0.3	10 mmol L^−1^	200	87.4%	3rd to 47%	582.67	47
N–Cu/biochar	0.2	0.5 mmol L^−1^	20	100%	3rd to 65%	100	48
Co/p-CN	0.2	0.4 g L^−1^	25	85.6%	—	107	49
Fe–Ce/DIA	0.02	10 mmol L^−1^	50	80%	3rd about 70%	2000	50
Ni_0.6_Fe_2.4_O_4_	0.35	42.0 μmol L^−1^	20	86%	4th to 83%	49.14	51
Co/BiFeO_3_	0.5	3.33 g L^−1^	10	81.09%	—	16.22	52
Ag_0.4_–BiFeO_3_	0.3	5 mmol L^−1^	10	91%	4th to 75%	30.33	53
Co_3_V_2_O_8_	0.2	40 mmol L^−1^	50	87.1%	4th about 60%	217.75	54
MnFe_2_O_4_	0.2	2 mmol L^−1^	25	36.34%	—	45.43	55
CuFe2O4@LBC	0.2	0.5 g L^−1^	25	85%	—	106.25	56
ACS-20	0.05	2 mmol L^−1^	50	87.4%	3rd to 24.6%	874	This work

a (mg of TC degradation per g of catalyst).

In an attempt to regenerate the deactivated ACS-20, it was heat treated (Ar atmosphere) at 350 °C and 600 °C for 2 hours. The results showed that the thermal regeneration treatment at 350 °C brought the TC degradation efficiency back up to 57.0%, while the thermal regeneration treatment at 600 °C further increased it to 72.1%. In order to explore the reason for deactivation, N_2_ isothermal adsorption–desorption and XPS characterization were performed on ACS-20 before and after the first cycle. As shown in Fig. 6b–d, after the first cycle, the specific surface area of ACS-20 was reduced from 889 m^2^ g^−1^ to 553 m^2^ g^−1^, the pore volume from 1.99 cm^3^ g^−1^ to 1.27 cm^3^ g^−1^, and the thiophene sulfur content from 82.4% to 77.6%. After regeneration by heat treatment at 600 °C, these parameters recovered to 820 m^2^ g^−1^, 1.31 cm^3^ g^−1^ and 80.0%, respectively. These results indicate that the pore structure of ACS-20 was severely blocked by adsorbed TC molecules and their intermediate products, resulting in a decrease in its adsorption performance and the number of effective active sites. Thermal regeneration treatment can effectively remove the blockage in the pores and re-expose some of the active sites, thus partially restoring the catalytic activity.

## 4. Conclusions

In summary, ACS catalysts with different pore structures were prepared *via* a nano-MgO template method using d(+)-glucose as the carbon source and sulfur as the sulfur source. Characterization results showed that the particle size of nano-MgO significantly affected the pore structure as well as the sulfur doping level of the ACSs, which in turn affected their adsorption performance and catalytic activity for PDS activation and TC removal. As a result, ACS-20 prepared with 20 nm nano-MgO as the template possessed the largest surface area and pore size and the highest sulfur content, which showed the best adsorption–catalytic performance toward TC removal. ACS-20 also presented outstanding performance over a wide pH range. A reaction mechanism investigation over ACS-20 indicated that both radical pathways and non-radical pathways participated in PDS activation, but the non-radical pathway with non-radical ^1^O_2_ and metastable PDS complexes as major oxidative species plays the dominant role in TC removal. A reusability and stability study showed that, although ACS-20 presented inferior reusability, it can be well recovered through thermal treatment. This work provides a promising strategy and some theoretical basis for the design and preparation of activated carbon catalysts for advanced oxidation reactions from the points of view of pore structure design and S-doping.

## Conflicts of interest

There are no conflicts to declare.

## Supplementary Material

RA-014-D3RA08958D-s001
