# Supplementary material for: Sulfur-doped activated carbon for the efficient degradation of tetracycline with persulfate: Insight into the effect of pore structure on catalytic performance
Source: RSC Adv. 2024 Apr 10;14(16):11470–81. doi: 10.1039/d3ra08958d (PMC11005904; doi:10.1039/d3ra08958d)
Supplement: RA-014-D3RA08958D-s001 [file RA-014-D3RA08958D-s001.pdf]

Electronic Supplementary Material (ESI) for RSC Advances.
This journal is © The Royal Society of Chemistry 2024

Supporting Information

Page 1 – Information on manuscript

Sulfur-doped activated carbon for the efficiently degradation of tetracycline with persulfate：Insight to the effect of pore structure on catalytic performance

Yaoping Guo[a], Yaxiong Huang[a], Yifan Li[a], Yan Luo[c], Keng Xuan[a, b*], Yadan Guo[a*], Hao Jiang[a] and Rui Fang[a]

[a]School of Water Resources Environmental Engineering, East China University of Technology, Nanchang 330013, China

[b]School of Surveying and Mapping and Spatial Information Engineering, East China University of Technology, Nanchang 330013, China

[c]Jiangxi Engineering Province Engineering Research Center of New Energy Technology and Equipment, East China University of Technology, Nanchang 330013, China

*Corresponding author. Tel.: +86 18734907983; E-mail: xuankeng@ecut.edu.cn (K. Xuan)

Page 2 – Content

Section S1. Experimental

Text S1. Measurement of the surface zero charge point of the ACS-20.

Disperse 250 mg of ACS-20 catalyst evenly into 4 mL of boiled and cooled deionized water, mix thoroughly and let stand. At a certain interval, the pH meter was used to measure the pH of the solution. When the pH of the solution does not change, the pH at this time is the surface point zero charge.

Section S2. Characterization of catalysts

S2.1. Textural properties of catalysts

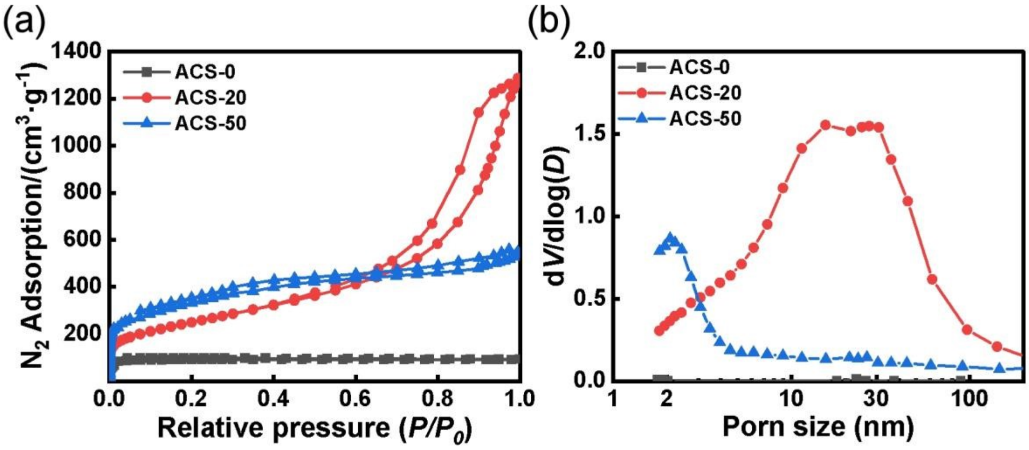

Fig. S1 N_2 sorption isotherms, and (e) pore size distributions of ACS-Z activated carbon.

S2.2. XPS analysis of catalysts

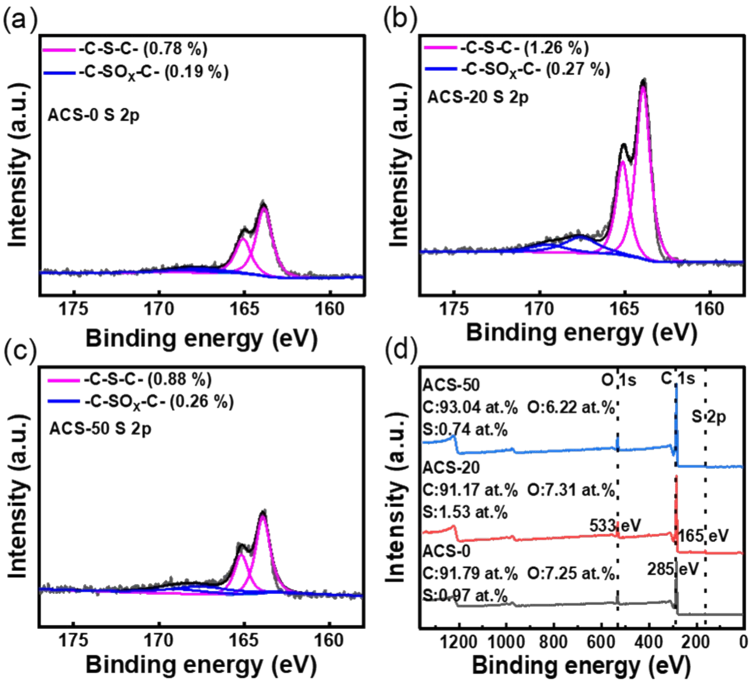

Fig.S2 The XPS S 2p spectra of (a) ACS-0, (b) ACS-20, (c) ACS-50 and (d) The XPS survey of ACSs.

S2.3. Adsorption properties of Catalysts

Table S1. Intra-particle diffusion kinetic model parameters for TC adsorption.

Catalysts	k_1/ [mg/(g·min$^{0.5}$)]	R^2	k_2/ [mg/(g·min$^{0.5}$)]	R^2
ACS-20	1.359	1	0.371	0.9254
ACS-50	0.463	1	0.2823	0.9567

S2.4. Surface functional groups of Catalysts

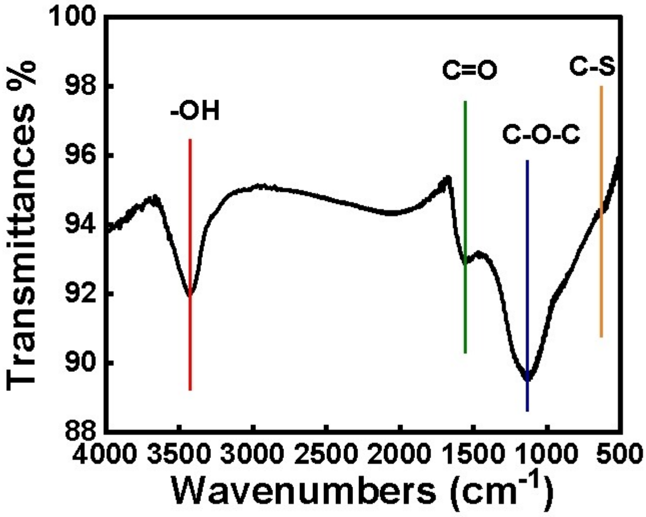

Fig.S3 The FT-IR spectrum of ACS of ACSs.

As shown in Fig S3, the peak at 620 cm^{-1} represents the C-S stretching vibration, and the peak at 3432 cm^{-1} corresponds to the hydroxyl characteristic peak. The antisymmetric stretching vibration absorption peak of $-COO^-$ and the C-O stretching vibration absorption peak were observed at 1565 cm^{-1} and 1135cm^{-1}, respectively. Based on the above analysis, it was found that the surface of ACS-20 contained the groups of $-COO^-$, -C-O-C- and -OH.
